# How healthy are chronically ill patients after eight years of homeopathic treatment? – Results from a long term observational study

**DOI:** 10.1186/1471-2458-8-413

**Published:** 2008-12-17

**Authors:** Claudia M Witt, Rainer Lüdtke, Nils Mengler, Stefan N Willich

**Affiliations:** 1 Institute for Social Medicine, Epidemiology and Health Economics, Charité University Medical Center, D-10098 Berlin, Germany; 2 Karl and Veronica Carstens-Foundation, Am Deimelsberg 36, D-45276 Essen, Germany

## Abstract

**Background:**

Homeopathy is a highly debated but often used medical treatment. With this cohort study we aimed to evaluate health status changes under homeopathic treatment in routine care. Here we extend former results, now presenting data of an 8-year follow-up.

**Methods:**

In a prospective, multicentre cohort study with 103 homeopathic primary care practices in Germany and Switzerland, data from all patients (age >1 year) consulting the physician for the first time were observed. The main outcome measures were: The patients' perceived change in complaint severity (numeric rating scales from 0 = no complaint to 10 = maximal severity) and quality of life as measured by the SF-36 at baseline, and after 2 and 8 years.

**Results:**

A total of 3,709 patients were studied, 73% (2,722 adults, 72.8% female, age at baseline 41.0 ± 12.3; 819 children, 48.4% female, age 6.5 ± 4.0) contributed data to the 8-year follow-up. The most frequent diagnoses were allergic rhinitis and headache in adults, and atopic dermatitis and multiple recurrent infections in children. Disease severity decreased significantly (p < 0.001) between baseline, 2 and 8 years (adults from 6.2 ± 1.7 to 2.9 ± 2.2 and 2.7 ± 2.1; children from 6.1 ± 1.8 to 2.1 ± 2.0 and 1.7 ± 1.9). Physical and mental quality of life sores also increased considerably. Younger age, female gender and more severe disease at baseline were factors predictive of better therapeutic success.

**Conclusion:**

Patients who seek homeopathic treatment are likely to improve considerably. These effects persist for as long as 8 years.

## Background

Homeopathy is based on the 'principle of similars', whereby substances that cause symptoms in healthy individuals are used to stimulate healing in patients who have similar symptoms when ill [1]. Usually, these substances are used in extremely high dilutions, which makes homeopathy a controversially debated system.

However, homeopathy is becoming increasingly popular in the world and constitutes an important factor of public health systems. For example, in the US the proportion of patients obtaining homeopathic care has quadrupled from 1991 to 1997 [2]. In the UK it was estimated that 2% had visited a homeopathic practitioner in the last 12 months [3] and that annual expenditures for homeopathy reached ₤34.04 million (out-of-pocket ₤30.74 million, NHS ₤3.3 million) [4]. In Germany, the country in which homeopathy originated, a survey demonstrated that approximately 10% of men and 20% of women in the general population used homeopathic medicines during the previous year [5]. Here the General Medical Council grants an official additional certification in homeopathy upon successful completion of a three-year-long training programme. This is held by approximately 4,500 physicians [6].

Meta-analyses of placebo controlled trials on homeopathy have shown inconsistent results [7-9]. However, there is only little data on the effectiveness and patients' satisfaction of homeopathic health care in everyday practice. Ten years ago we started a cohort study in nearly 4.000 patients aiming to systematically collect data about diagnoses and treatment in the area of homeopathic health care in Germany, including data on the patients' health status. Our first results, based on a two year follow-up, were published some years ago [10]. This paper extends our former report, now for the first time presenting data 8 years after the primary homeopathic treatment.

## Methods

### Study design

In this prospective multi-centre cohort study, patients were included consecutively upon their first consultation with a participating homeopathic physician. All study physicians hold an additional certification in classical homeopathy and had at least three years of experience in its practice. No restrictions on diagnoses were made. For details on inclusion criteria or on the selection of physicians see [10]. Recruitment period was between September 1997 and December 1999, and measurements of health status were taken at 3, 12, and 24 months using standardised questionnaires. The study protocol was approved by the ethics review board of the Charité University Medical Center. In total 3,981 (2,851 adults, 1,130 children) were originally included in the study.

In 2006 3,677 patients (2,603 adults, 1,074 children) were contacted again to provide an 8-year follow-up. Patients were not contacted if they were known to have deceased (32 adults), had withdrawn their consent to participation in this study (207 adults, 53 children) or their actual place of residence could not be identified (9 adults, 3 children).

In this paper we present only the long-term results (8 years), for more details on earlier time points refer to [10].

### Outcome measures

Standardized questionnaires were designed to document sociodemographic data, as well as information on prior medical history, patient symptoms and complaints, quality of life, and the use of any treatment other than homeopathy. At study entry, all patients recorded the complaints that led them to consider homeopathic treatment, for children below the age of 8 their parents were asked to do so. Independently of their physicians, patients rated the severity of their complaints on a numeric rating scale (NRS, 0 = no complaints, 10 = maximum severity). All complaints listed by patients in their baseline questionnaire were transferred to their follow-up questionnaires by the study office personnel. This ensured that each baseline complaint was assessed at each subsequent follow-up. For statistical purposes we averaged the ratings of the first four listed complaints and used this average as the main outcome measure.

For adults (16 years or older at study entry), general health-related quality of life (QoL) was assessed using the German MOS SF-36 questionnaire [11]. The results of the SF-36 are presented in normalised scores, the results being scaled in such a way that the normal German population has a mean score of 0 and a standard deviation of 1.

The first questionnaire was distributed to the patients by the study physician and completed prior to case taking and the start of therapy (baseline). Patients sent their completed questionnaires to the study office in sealed envelopes. Follow-up questionnaires were sent to all patients by the study office.

At the 8-year follow-up we additionally measured the overall patient satisfaction with treatment on a 4-point Likert scale, ranging from 1 ("little satisfied") to 4 ("very satisfied"). Moreover, we asked the patients to rate whether they "would let their disease be treated homeopathically again", "would try homeopathy in other diagnoses", "would recommend homeopathy to a friend", and "find homeopathy logically comprehensible", each on a NRS (0 = "I totally disagree" 10 = "I absolutely agree").

All patients were asked whether they were still under homeopathic treatment. If not, the specific reasons for stopping treatment were recorded and classified into (1) "treatment successful", including "complete healing" and "major improvement"; (2) "treatment success unsatisfactory", including "unsatisfactory patient-physician relationship" "hospitalisation", "treatment not helpful", "deterioration", "other therapies preferred", and "too long distance"; (3) "unrelated to treatment success", including "physician deceased or retired", "physician or patient moved", "pregnancy", "limited time", "treatment too expensive". In case of multiple answers we assumed "treatment success unsatisfactory" if only one of the above listed reasons was given, regardless what the other reasons were.

### Treatments

To reflect usual care all physicians were completely free to choose a treatment. This usually included the prescription of homeopathic medicines according to homeopathic principles, but also could include the onset, change, or withdrawal of a conventional medicine, referrals to specialists, or admission to a hospital.

At the 8-year follow-up all patients were asked to specify which, if any, complementary or conventional therapies they used besides homeopathy. For reasons of clarity we grouped these therapies into non-homeopathic medical therapies (phytotherapy, Chinese herbal medicine), relaxation therapies (meditation, autogenous training), energetic therapies (bioresonance therapy, reiki, shiatsu, kinesiology, Feldenkrais), exercise therapies (yoga, tai chi, qigong), and manual therapies (osteopathy, cupping).

### Statistics

Statistical analysis (using SAS/STAT© 9.1 software) followed the intention-to-treat principle and included those 3,709 patients (2,635 adults, 1074 children) who were contacted at the 8-years follow-up or were known to have deceased.

If patients reported that their complaints were cured we replaced missing values with a severity = 0 in subsequent records. Deceased patients were assigned a severity = 10. The remaining missing values were multiply imputed according to Rubin [12]: Each was given several plausible values (drawn from a multivariate normal distribution), generating a total of 5 distinct complete data tables, each without any missing value. These were analyzed separately (see below), and the results pooled to calculate treatment effects and p-values.

For each imputed data set, we fitted a generalised multiple linear regression model to the data [13], where time was taken as a three-level (baseline, 2 years, 8 years) within-patient factor and the serial correlation was assumed to be exponential with time. For comparability purposes with other studies we divided the estimated mean changes from this model by the standard deviation at baseline. This standardised mean change also allows assessing the clinical relevance of effects.

Moreover, we aimed to identify factors that predict treatment success. For this, we dichotomised the change of complaint severity at a cut point of 2 pts: improvements of 2 pts or more were defined as a clinically relevant success, smaller improvements or deteriorations were defined as inadequate success. A 2-point improvement in the NRS approximately represents the improvement of one standard deviation at baseline and can thus be seen as a threshold of clinical relevance. At first a list of potential predictors was compiled. This list included mean severity of complaints at study entry, age at study entry (linear factors), sex, the most frequent diagnoses at study entry (migraine, tension type headache, sleep disorders, depression, anxiety disorders, multiple eczemas, psoriasis, allergic dermatitis, allergic rhinitis, allergies, dysmenorrhea, multiple infections, hypertension, low back pain, asthma), concomitant therapies (conventional medicine, anthroposophic medicine, acupuncture, other TCM therapies, phytotherapy, osteopathy, other manual therapies, yoga, other exercise therapies, relaxation therapies, naturopathy), additional visits to other doctors (conventional, TCM, anthroposophic, naturopathic), hospital admission, and reasons for stopping treatment (treatment successful, treatment success unsatisfactory). Afterwards, predictors were identified by backward selection in a logistic regression model.

Data for adults (>16 years at study entry) and children (<16 years) were analysed separately.

## Results

### Response rates and basic characteristics

In total 2,722 (1,903 adults, 819 children) contributed data to the 8-years follow-up. Patients in this study suffered from long-term chronic diseases (table 1). Response rates were considerable higher in female than in male adults (74.3% vs. 67.2%) but similar in female and male children (76.9% vs. 75.7%). Thus, male adults are somewhat a bit underrepresented in our sample. Age at study entry matched the data of the complete sample (table 1).

**Table 1:** Patient characteristics (values are absolute numbers and percent or mean ± standard deviation)

	Study population		Responders only	
	Adults (n = 2,635)	Children (n = 1,074)	Adults (n = 1,903)	Children (n = 819)

Sex (male: female)	771:1864	559:515	518:1385	423:396
Age at study entry (years)	40.6 ± 12.4	6.7 ± 4.1	41.0 ± 12.3	6.5 ± 4.0
Age at 8-year follow-up (years)	48.3 ± 12.4	14.2 ± 4.2	48.8 ± 12.3	14.1 ± 4.2
Marital status (living in partnership)	1916 (72.7%)		1405 (73.9%)	
Education (attending school >10 years)	1570 (59.6%)		1155 (60.7%)	
Belief in homeopathy at study entry	1744 (66.2%)	739 (68.8%)	1283 (67.4%)	567 (69.2%)
Duration of disease at study entry (years)	10.0 ± 9.6	4.3 ± 2.7	9.8 ± 8.7	4.2 ± 3.5
Intake of conventional drugs at study entry	1318 (50.0%)	340 (31.7%)	965 (50.7%)	273 (33.3%)
Primary diagnosis at study entry *				
Allergies (ICD9: 995.3)	154 (5.8%)	65 (6.1%)	114 (6.0%)	51 (6.2%)
Anxiety (ICD9: 300.0)	137 (5.2%)	44 (4.1%)	94 (4.9%)	34 (4.2%)
Asthma (ICD9: 493.9)	109 (4.1%)	67 (6.2%)	88 (4.6%)	51 (6.2%)
Depression (ICD9: 311.0)	157 (6.0%)	5 (0.5%)	110 (5.8%)	2 (0.2%)
Eczema (ICD9: 692.9)	200 (7.6%)	48 (4.5%)	154 (8.1%)	42 (5.1%)
Multiple infections (ICD9: 796.6)	140 (5.3%)	183 (17.0%)	105 (5.5%)	141 (17.2%)
Migraine (ICD9: 346.9)	202 (7.7%)	16 (1.5%)	146 (7.7%)	12 (1.5%)
Atopic dermatitis (ICD9: 691.8)	131 (5.0%)	216 (20.1%)	99 (5.2%)	175 (21.4%)
Allergic rhinitis (ICD9: 477.9)	215 (8.2%)	58 (5.4%)	161 (8.5%)	45 (5.5%)
Headache (ICD9: 784.0)	216 (8.2%)	71 (6.6%)	155 (8.1%)	45 (5.5%)
Sleep disorders (ICD9: 780.5)	185 (7.0%)	77 (7.2%)	127 (6.7%)	58 (7.1%)

* Multiple diagnoses allowed

The majority of the patients were highly educated female adults, most of them fairly below the age of 60 (table 1). Adults mainly suffered from headache (tension type and migraine), allergic diseases, or skin diseases, children from atopic eczema or multiple infections. The average number of diseases at baseline was 2.8 ± 1.1 in adults and 2.3 ± 1.0 in children.

### Treatments

Eight years after study entry one third of the patients (n = 897, 32.9%) still were under homeopathic treatment. 657 patients (24.1%) still consulted that homeopathic physician they had chosen at study entry, 240 (8.8%) had changed to another homeopath. Three in ten patients had stopped homeopathic treatment because they perceived major improvements of health status (n = 794, 29.2%). On the other hand, a similar percentage of patients stopped treatment because they did not feel homeopathy could help them sufficiently (n = 708, 26.0%), including those 42 patients (1.5%) who reported a deterioration. 194 patients (7.1%) said they stopped treatment for reasons unrelated to the therapy success (moving, financial shortage, physician retired etc.), 97 (3.6%) did not give any reason. These figures differed considerably between adults and children: the percentage of children who stopped treatment because of major improvements was twice that of adults (n = 378 (46.2%) vs. n = 416 (21.9%)). In contrast, adults more often stopped treatments because of perceived treatment failure (n = 567 (29.8%) vs. n = 141 (17.2%)).

Nearly half of the patients (n = 1118, 41.1%) reported to have consulted another CAM therapist (not homeopathic) during the study period, including naturopathic doctors, physicians for Traditional Chinese Medicine, and non-medical therapists (German "Heilpraktiker"). Four in ten patients were treated with conventional remedies, this rate being considerably higher in adults than in children (table 2). Similarly, children used less frequently other CAM therapies (table 2). Differences between those who stopped homeopathic treatment and those who continued were small in children. However in adults patients who stopped treatment used more frequently conventional medication (53% vs. 38%).

**Table 2:** Number of patients receiving non-homeopathic treatments during the last 5 years of follow-up, grouped whether or not they still were under homeopathic treatment

	Adults			Children		
	total	still under treatment	treatment stopped	total	still under treatment	treatment stopped

Conventional medicines	881* (46.3%)	255 (37.6%)	625 (52.5%)	154 (18.8%)	40 (18.3%)	114 (19.0%)
Acupuncture	402 (21.1%)	155 (22.9%)	247 (20.8%)	38 (4.6%)	8 (3.7%)	30 (5.0%)
Yoga	181 (9.5%)	79 (11.7%)	102 (8.6%)	10 (1.2%)	6 (2.7%)	4 (0.7%)
Relaxation therapies^#^	176 (9.2%)	56 (8.3%)	120 (10.1%)	13 (1.6%)	2 (0.9%)	11 (1.8%)
Energetic therapies^#^	188 (9.9%)	68 (10.0%)	120 (10.1%)	56 (6.8%)	20 (9.1%)	36 (6.0%)
Exercise therapies^#^	249 (13.1%)	109 (16.1%)	140 (11.8%)	11 (1.3%)	6 (2.7%)	5 (0.8%)
Manual therapies^#^	108 (5.7%)	53 (7.8%)	55 (4.6%)	16 (2.0%)	9 (4.1%)	7 (1.2%)
Non-conventional medicines^#^	60* (3.2%)	15 (2.2%)	44 (3.7%)	6 (0.7%)	2 (0.9%)	4 (0.7%)

* Due to missing values frequencies in subgroups do not add to total frequencies

# For definitions see methods section

### Severity of complaints and quality of life

**Figure 1:**
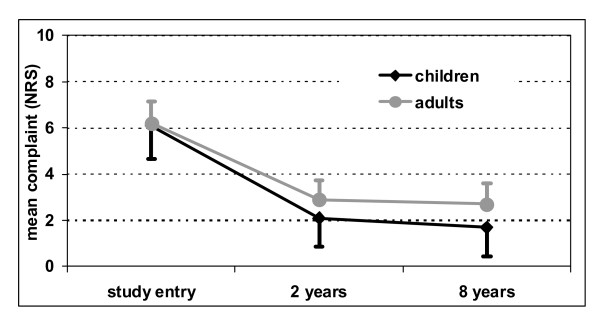
Severity of complaints (mean ± standard deviation).

During the study mean severity of complaints improved from baseline 6.2 ± 1.7 to 2.7 ± 2.1 after 8 years in adults and from 6.1 ± 1.8 to 1.7 ± 1.9 in children (table 3, figure 1). From the generalised linear model the respective standardised mean changes (mean changes divided by standard deviations at baseline) were estimated at 1.61 for adults (CI: 1.54 to 2.68, p < 0.001) and 2.01 for children (CI: 1.89 to 2.12, p < 0.001).

**Table 3:** Course of mean complaint severity and quality of life during the study, grouped whether or not the patients still were under homeopathic treatment

	baseline	2 years	8 years	2 years change	8 years change
Adults					

Severity of complaints					
total	6.2 ± 1.7	2.9 ± 2.2	2.7 ± 2.1	3.2 ± 2.4	3.5 ± 2.4
still under hom. treatment	6.0 ± 1.6	2.6 ± 1.9	2.4 ± 1.9	3.4 ± 2.3	3.6 ± 2.2
hom. treatment stopped	6.2 ± 1.8	3.0 ± 2.3	2.8 ± 2.2	3.2 ± 2.4	3.4 ± 2.5
Quality of life, physical score					
total	-0.36 ± 0.96	0.08 ± 0.85	0.08 ± 0.89	0.42 ± 0.91	0.41 ± 1.00
still under hom. treatment	-0.34 ± 0.92	0.16 ± 0.79	0.15 ± 0.81	0.48 ± 0.89	0.48 ± 0.95
hom. treatment stopped	-0.37 ± 0.39	0.04 ± 0.89	0.04 ± 0.93	0.41 ± 0.91	0.38 ± 1.02
Quality of life, mental score					
total	-1.47 ± 1.43	-0.56 ± 1.23	0.53 ± 1.26	0.87 ± 1.41	0.95 ± 1.51
still under hom. treatment	-1.43 ± 1.44	-0.49 ± 1.19	-0.43 ± 1.17	0.89 ± 1.41	1.00 ± 1.52
hom. treatment stopped	-1.50 ± 1.43	-0.61 ± 1.24	-0.59 ± 1.31	0.86 ± 1.48	0.92 ± 1.51

Children					

Severity of complaints					
total	6.1 ± 1.8	2.2 ± 2.0	1.7 ± 1.9	3.9 ± 2.5	4.4 ± 2.6
still under hom. treatment	6.1 ± 1.7	2.1 ± 1.9	1.8 ± 1.9	4.0 ± 2.4	4.3 ± 2.4
hom. treatment stopped	6.1 ± 1.8	2.2 ± 2.1	1.7 ± 1.9	3.9 ± 2.6	4.4 ± 2.6

At the 8-years follow-up one in two patients reported improvements of complaint severity by 50% or more. These percentages were similar in patients who were still under homeopathic treatment and those who were not (table 4).

**Table 4:** Number of patients whose change in symptom scores indicates improvements or worsening of complaints at the 8-years follow-up, grouped whether or not they still were under homeopathic treatment

	Adults n (%)			Children n (%)		
	total	still under treatment	treatment stopped	total	still under treatment	treatment stopped

cured (all complaints vanished)	243 (12.8%)	86 (12.7%)	157 (13.2%)	247 (30.2%)	62 (28.3%)	185 (30.9%)
complaint severity improved ≥ 50%	918 (48.2%)	372 (54.9%)	545 (45.8%)	397 (48.5%)	111 (50.7%)	286 (47.7%)
complaint severity improved ≥ 10%	488 (25.6%)	162 (23.9%)	326 (27.4%)	121 (14.8%)	31 (14.2%)	90 (15.0%)
complaint severity worsened ≥ 10%	90 (4.7%)	21 (3.1%)	69 (5.8%)	32 (3.9%)	8 (3.7%)	24 (4.0%)

Accordingly, QoL in adults improved considerably (table 3). This results in effect size estimates of 0.39 (CI: 0.35 to 0.45, p < 0.001) in the physical score and 0.54 (CI: 0.48 to 0.60, p < 0.001) in the mental score, respectively.

These 8-year figures were nearly identical to those after 2 years (table 3) suggesting that the patients' health status did not worsen along time. Again, in children there were no relevant differences between those who stopped homeopathic treatment and those who continued, whereas in those adults who continued treatment we found slightly higher effects.

### Overall satisfaction

731 (38.4%) adults and 342 (41.8%) children reported to be "very satisfied" with the treatment, in contrast only 246 (12.9%) adults and 84 (10.3%) children were "little satisfied". Accordingly, most patients would use homeopathy again and recommend it to friends with similar complaints (table 5).

**Table 5:** Patients' assessments of homeopathic treatment (each scale assessed on a NRS from 0 = I totally disagree to 10 = I absolutely agree; mean ± standard deviation)

	Adults	Children
"I would let my disease be treated again homeopathically"	7.5 ± 3.2	7.7 ± 3.0
"I would recommend homeopathy to my friends"	7.7 ± 3.0	7.8 ± 2.9
"I would use homeopathy with other diseases"	8.2 ± 2.6	8.0 ± 2.5
"I find homeopathy logically comprehensible"	7.3 ± 2.8	6.5 ± 2.8

### Predictors of success

1283 adults (67.4% of the study population, 48.7% of all responders) and 655 children (80.0%/61.0%) experienced a clinically relevant treatment success, defined as an improvement of complaint severity of 2 pts or more. From the logistic regression we found that this was more likely in women than in men, and in children than in adults. Patients who simultaneously used other treatments (conventional or complementary) had a smaller chance to improve relevantly, as did those suffering from allergies, allergic rhinitis, or headache. In contrast, a diagnosis of multiple infections was a positive predictor (table 6).

**Table 6:** Prediction of treatment success (mean complaint improvement >2 pts on a NRS from 0–10) from a logistic regression analysis

Predictor	Odds-ratio	p-value
Mean complaint at baseline (each pt)	1.74 (1.63 to 1.85)	<.001
Age (each 10 years)	0.83 (0.78 to 0.88)	<.001
Men (vs women)	0.73 (0.59 to 0.89)	0.003
Additional non-homeopathic co-medication	0.46 (0.37 to 0.56)	<.001
Additional treatment at naturopath	0.72 (0.57 to 0.90)	0.003
Additional treatment: Cupping	0.46 (0.23 to 0.91)	0.025
Additional treatment: Osteopathy	0.63 (0.38 to 1.06)	0.081
Diagnosis: allergy (ICD9: 995.3)	0.63 (0.43 to 0.91)	0.014
Diagnosis: allergic rhinitis (ICD9: 477.9)	0.66 (0.47 to 0.92)	0.013
Diagnosis: multiple infections (ICD9: 796.9)	1.60 (1.09 to 2.34)	0.016
Diagnosis: headache (ICD9: 784.0)	0.68 (0.48 to 0.97)	0.033

## Discussion

In our study we extended former results on the course of disease in patients receiving homeopathic treatment, now presenting data from an 8-year follow-up. These data consistently show substantial health improvements in patients under homeopathic treatment, which persisted through the whole observation period. Improvements were more pronounced in younger patients, females, and those with greater disease severity at baseline.

The methodological strengths of our study include consecutive enrolment of a large sample size, the participation of approximately 1% of all physicians certified to practice homeopathy in Germany and the use of standardised outcome instruments also used in studies on conventional therapy.

Moreover, our study provides a reasonably representative sample of all patients attending a doctor practicing classical homeopathy in Germany. The subset of patients responding to the 8-year follow up matched fairly well the data of the complete sample: although female adults were slightly overrepresented in this sample, data on age, complaint severity at baseline or duration of disease were nearly identical between those who responded after 8 years and those who did not. We therefore believe that selection bias is small and that our data are generalisable.

Our study was designed to evaluate homeopathic treatment in patients with various multiple diagnoses. This disallowed the use of disease-specific measurement instruments. Instead we used a numeric rating scale which is validated, often used [14] and allowed for assessments of a specific complaint as well as for generalization and interpretation across various diagnoses. Using generic QoL questionnaires served the same purpose.

As patients were allowed to use conventional therapies and other complementary therapies during the study period, the observed improvements cannot be attributed to homeopathic treatment alone. The aim of this study, however, was not to test the effectiveness of homeopathic drug treatment, but rather provide an unbiased representation of contemporary homeopathic health care and its outcome in routine care.

The mean change of the severity ratings after 8 years was large. This may be partly explained by placebo and/or regression to the mean effects that our study was not designed to control. We thus cannot rule out overestimation of the treatment effect. The QoL improvements, on the other hand, may have been greater than recorded: The SF-36 is unlikely to overestimate changes, its mental scales have been found to be less sensitive than the mental und social scales of other instruments such as the Duke Health Profile [15]. It is most unlikely that regression to the mean accounts for all QoL improvement that we have described: on the physical scale the adults scored even better than the average German population. Moreover, patients in this study suffered from long-term chronic diseases and nearly all of them were conventionally pretreated [10]. This strengthens the likelihood that the improvement is not purely due to the natural history of the condition.

It is of note that the differences in the outcome between those patients who stopped treatment and those who still continued were small. Most patients reported improvements and only 5% of patients stopped treatment because of aggravations.

Moreover, only few diagnoses turned out as a predictive factor for treatment success. This might be taken as an indicator that the difference in outcome was similar for most diagnoses and that diagnosis was not a factor severely confounding our results.

Patients who used additional treatments had a worse outcome than those who did not. This presumably does not reflect the fact that these treatments were ineffective or even harmful, but is more likely a consequence from self-selection: patients who did not benefit from the homeopathic treatment are more likely to seek additional treatment.

To our knowledge, the present study is the first to evaluate systematically health effects under homeopathic treatment for such a long observation period and with a high follow-up rate. Güthlin et al., for example, investigated 933 chronically ill German homeopathy patients for a period of 30 months (only 129 providing data at that time point) and found comparable QoL effect sizes [16]. In England, Spence et al. followed over 6.500 patients from a single homeopathic outpatient unit for an individual time period (maximum 6 years, average unknown) [17]. Using a 7-point Likert scale of global clinical impression as an outcome measure they estimated that about 50% of all patients showed relevant improvements, a figure that matches our estimates. Several other investigations from different countries in Europe or America report similar health effects in various diseases within the first year after homeopathic treatment. Here the percentages of patients who experienced substantial improvements were consistently above 50%, [15,18-28], although conventional medication was reduced [20,23,24].

## Conclusion

Our findings demonstrate that patients who seek homeopathic treatment are likely to improve considerably, although this effect must not be attributed to homeopathic treatment alone. These effects persisted for 8 years.

## Competing interests

The authors declare that they have no competing interests.

## Authors' contributions

CMW was substantially involved in the conception and design of the study, supervised it, helped to interpret the data, and revised the manuscript critically. RL was responsible for the analysis and interpretation of data and wrote the first draft of the manuscript. NM was responsible for data acquisition, helped to interpret the data and revised the manuscript critically. SNW was substantially involved in the conception and design of the study, acquired funding, and revised the manuscript critically. All authors read and approved the final manuscript.

## Pre-publication history

The pre-publication history for this paper can be accessed here:


